# An Analysis of the Governance, Ethical, Legal, and Social Implications of Biocomputing

**DOI:** 10.1007/s11948-025-00572-x

**Published:** 2026-01-02

**Authors:** Renée A. Sirbu, Luciano Floridi

**Affiliations:** 1 Yale Digital Ethics Center, Yale University, 85 Trumbull Street, New Haven, CT 06511 USA; 2 Department of Legal Studies, University of Bologna, Via Zamboni 22, 40100 Bologna, Italy

**Keywords:** Biological data ownership, Biocomputing, Ethical implications, Governance frameworks, Regulatory oversight

## Abstract

Biological computing (biocomputing) leverages biologically derived materials and processes, such as DNA and protein synthesis, to perform computational tasks. Biocomputing offers significant advantages over traditional silicon-based systems in terms of scalability, energy efficiency, computational flexibility, and information storage potential. However, the distinct operational characteristics of biocomputing raise novel governance, ethical, legal, and social implications (GELSI). This article identifies and analyzes key GELSI concerns raised by biocomputing. It then highlights the inadequacies of current regulatory frameworks in addressing the unique challenges posed by massively parallel molecular computations, biosafety risks, probabilistic error management in clinical applications, patentability of biological storage systems, and ownership rights in self-replicating data systems. The analysis concludes by recommending specialized regulatory approaches and international collaboration to govern biocomputing technologies responsibly, ensuring their ethical integration and equitable benefits across global societies.

## Introduction

Biological computing, or *biocomputing,* uses biologically derived materials (e.g., DNA) and processes (e.g., protein synthesis and translation) to perform computational tasks, such as informational processing or solving mathematical problems. By using molecular pathways to generate outputs (i.e., solutions to computational tasks) based on specific inputs (i.e., engineered conditions), biocomputers can operate via several signalling pathways. The four most common are *biochemical*, producing molecular outputs, like chemical compounds; *biomechanical*, producing physical outputs, like locomotion; *bioelectronic*, producing electrical signalling outputs, like neural synapses; and *network-based*, producing consensus via independent cellular responses. A promising frontier in modern computing science, biocomputing has the potential to perform complex technical tasks, offering new solutions to computational limitations faced by traditional silicon-based systems, including proportionally higher computational throughput and greater flexibility than conventional computers. At the same time, biocomputing may also present unique governance, ethical, legal, and social implications (GELSI) that warrant careful consideration.

Our investigation reveals several GELSI that demand increased regulatory attention, particularly regarding scalability, energy efficiency, error rate, and information storage potential. We selected these four characteristics because they are distinctive operational features of biocomputing that differentiate it from traditional computing systems, each with substantial implications for governance frameworks. We do not discuss broader classical digital challenges—such as privacy, transparency, and accountability—as they apply universally across digital technologies and are not unique to biocomputing. Throughout this analysis, we also explore whether regulatory frameworks have been proposed to understand and categorize the identified GELSI.

Ultimately, biocomputing demands an interpretive shift in the GELSI paradigm. Unlike digital technologies or synthetic biology, which often rely on pre-existing, fixed ethical taxonomies (e.g., data privacy, biosecurity, fairness), biocomputing challenges the very ontological distinction between medium and message. In this context, GELSI must be reconceived not as a fixed checklist but as a dynamic interface where biology and information mutually redefine each other’s governance needs. Thus, the framework advanced here treats GELSI in biocomputing as a function of four entangled conditions: molecular unpredictability, evolutionary mutability, hybrid accountability, and infrastructural asymmetry. Each section applies this interpretive approach to assess not merely the presence of risks, but the reconfiguration of moral and legal categories introduced by biological computation. To this end, the rest of the article is structured into five more sections. In Section “Scalability in Biocomputing: Verification Challenges and Biosafety Considerations”, we begin our discussion of the four key characteristics of biocomputing by analyzing scalability through the GELSI framework, emphasizing governance and ethical implications. In Section “Energy Efficiency in Biocomputing: Environmental Impact and Resource Democratization”, we expand our analysis to address energy efficiency, focusing on ethical and social implications. In Section “Error Rate in Biocomputing: Accuracy Metrics and Clinical Implications”, we shift to analyzing the potential error rate introduced by biocomputing, considering solely its governance implications. In Section “Information Storage in Biocomputing: Patentability, Data Ownership, and Regulatory Oversight”, we offer one final discussion of the unique GELSI inherent to biocomputing by describing the information storage potential of these systems and identifying some legal implications. Finally, in Section “Conclusion and Future Considerations”, we conclude by synthesizing these insights and considering potential pathways forward for the responsible oversight of this field.

## Scalability in Biocomputing: Verification Challenges and Biosafety Considerations

Biocomputing systems achieve unprecedented computational scale through molecular parallelization, and gains in scale at low power are considered a significant advantage of biocomputing over traditional computing. However, this massive scalability introduces critical governance gaps around computational verification and safety: the parallel processing capabilities that make biocomputing so powerful simultaneously render traditional verification methods obsolete. Unlike electronic systems, wherein operations proceed sequentially and can be verified stepwise, the billions of simultaneous molecular interactions characteristic of biocomputers create a fundamental governance challenge.

In this section, we analyze the governance gaps that emerge when billions of molecular operations occur simultaneously and the ethical implications of generating large-scale biological systems for computational purposes. In “Verification challenges”, we use Leonard Adleman’s seminal biochemical DNA computer to illustrate this paradigm; readers familiar with this experiment may choose to skip this illustration. Our analysis focuses on two critical concerns: the near impossibility of monitoring parallel molecular computations and the biosafety implications of scaling bacterial populations for computational purposes.

### Verification Challenges

Molecular parallelization challenges existing governance frameworks for monitoring and verifying computational systems. Traditional software monitoring employs sequential “checks and balances,” whereby each operation is verified before the next operation is allowed to proceed (Banks, 2003; Maxim & Kessentini, 2016). The verification mechanisms that work effectively for sequential electronic computing fail when applied to the massively parallel operations characteristic of biocomputing. Conventional verification depends on tracing each computational step as it occurs; the simultaneous execution of billions of molecular reactions fundamentally undermines this approach, making operation-by-operation validation technically infeasible and conceptually inappropriate for these biological systems (Wittenstein et al., 2022).

**Fig. 1:**
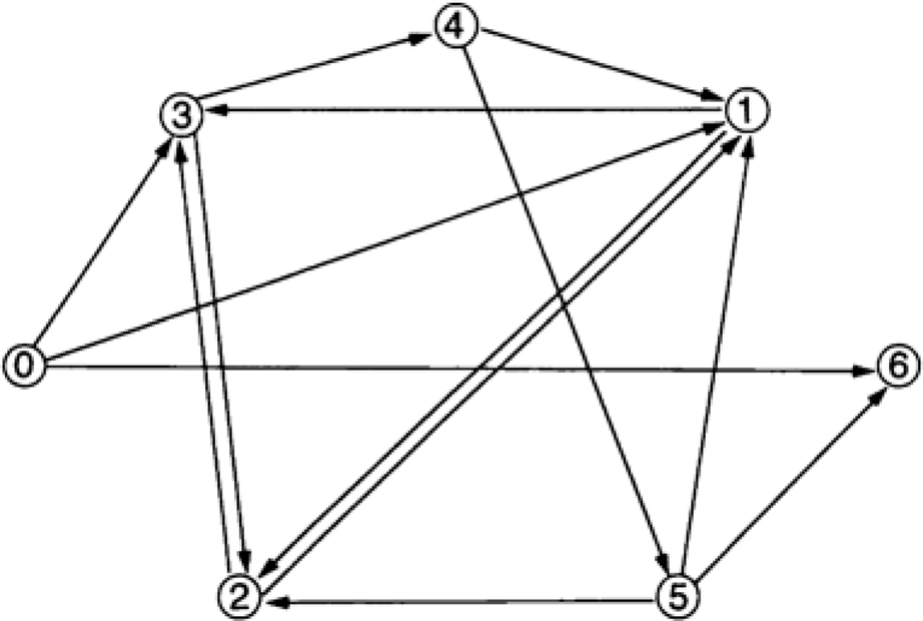
Graph from Adleman (1994) with designated vertices *v*_in_ = 0 and *v*_out_ = 6 for which a Hamiltonian (0→1, 1→2, 2→3, 3→4, 4→5, 5→6) path exists (Adleman, 1994)

For example, in 1994, Adleman used a human-operated DNA computer to solve a six-vertice directed Hamiltonian path problem (see Fig. 1).

Adleman encoded graph vertices as DNA sequences and edges as partial sequence overlaps. He then used DNA ligation to create potential paths, which he amplified using polymerase chain reaction (PCR) mechanisms to select valid solutions (Adleman, 1994). Through these molecular processes, Adleman’s DNA computer executed the following algorithm to prove that a Hamiltonian path exists for this problem:

- *Step 1:* Generate random paths through the graph.
- *Step 2:* Keep only paths that begin with *v*_in_ and end with *v*_out_.
- *Step 3:* If the graph has *n* vertices, then keep only paths that enter exactly *n* vertices.
- *Step 4:* Keep only paths that enter all of the vertices of the graph at least once.
- *Step 5:* If any paths remain, say “Yes”; otherwise, say “No.”

Adleman’s experiment was the first instance of using biomolecules to compute complex mathematical operations, establishing biochemical computing as a nascent field and outlining some of its major advantages over traditional computing.

Despite its computational power, no practical method has since been developed to individually validate each of the 10^14^ simultaneous ligation operations required by Adleman’s computer—if it did, any such method would be time-consuming and error-prone. Furthermore, molecular operations occur at nanoscale dimensions, where direct observation and real-time monitoring of individual molecular operations remain challenging even with advanced microscopy. Thus, molecular computing lacks an equivalent formal verification framework due to its inherently stochastic mechanism of action and microscopic dimensionality. This verification gap introduces significant governance concerns regarding reliability and safety. Biomolecular computing systems may require probabilistic accuracy metrics rather than deterministic guarantees; however, practical implementation is still lacking.

Biochemical computers are not alone in their verification challenges; all biocomputing architectures introduce obstacles to developing new probabilistic governance frameworks. For example, bioelectronic computing research has shown that neurons act as multiple independent threshold units. However, as Sardi et al. show, neurons also possess significant parallel processing capabilities, suggesting that bioelectronic systems could, in fact, scale in two ways: first, by increasing the number of individual neurons and second, by leveraging the inherent parallel processing abilities of each neuron at scale (Sardi et al., 2017). Thus, verification mechanisms for bioelectronic systems would have to account for their potential to scale in multiple dimensions.

Due to these challenges, biocomputing introduces a new set of “black box” verification problems that complicate existing regulatory oversight and quality assurance. Any operational oversight is further amplified if these independent units are networked or act in tandem, as in some bioelectronic architectures. Potential verification solutions might include statistical sampling approache null (Genot et al., 2013) or the development of molecular “witness” systems that produce verifiable byproducts proportional to computational accuracy (Qian and Winfree, 2011; Thubagere et al., 2017). Related fields such as quantum computing or synthetic biology may lend their verification schemas; barrier certificates (Prajna et al., 2007) or composability strategies (Chalk et al., 2021) can be adapted to fit stochastic verification in biocomputing. However, verification frameworks adapted from quantum computing or synthetic biology might be insufficient if they fail to address the unique dynamism of biological systems, such as evolutionary adaptation and epigenetic variability. Future governance frameworks might benefit from explicitly considering adaptive verification methodologies that dynamically monitor biological states and computational integrity over time. Furthermore, leveraging machine learning algorithms for predictive verification could provide anticipatory oversight and real-time anomaly detection, strengthening safety protocols and enabling more precise probabilistic accuracy metrics.

### Biosafety Considerations

The scaling potential of biomechanical computing and network-based biocomputing introduces biosafety challenges for large bacterial populations. As Woodhouse and Dunkel show, biomechanical computing may be scalable through the coordinated movement of self-propelled biological agents (Woodhouse & Dunkel, 2017). Biomechanical systems leverage the inherent ability of active particle populations (e.g., bacteria or engineered motile colloids) to perform parallel computations through their collective patterns of motion. In the case of network-based biocomputing, both Tamsir and Didovyk’s experiments showed scaling potential through networked cellular components in distributed bacterial cell cultures (Didovyk et al., 2015). These systems possess higher computational power by multiplying bacterial networks, wherein each cell performs local computations that are pooled to contribute to a final solution.

Large-scale bacterial populations commonly used in biocomputing applications surpass typical laboratory scale (NIH, 2024), creating novel biosafety risks inadequately addressed by existing guidelines like the NIH Guidelines for Research Involving Recombinant or Synthetic Nucleic Acid Molecules. While these frameworks address containment at standard experimental scales, the magnitude required by biocomputing significantly intensifies biosafety concerns such as accidental environmental release, horizontal gene transfer, and ecological disruption. Both industry and academic stakeholders have highlighted this gap in available guidance (Church et al., 2014). Addressing these gaps necessitates developing targeted regulatory guidelines specifically calibrated for biocomputational bacterial populations, including clearly defined standards for secure containment, real-time monitoring, contingency protocols for breach scenarios, and training requirements for laboratory personnel to ensure rigorous biosafety compliance (Schmidt & de Lorenzo, 2012).

## Energy Efficiency in Biocomputing: Environmental Impact and Resource Democratization

Amid growing concerns about the environmental footprint of the technology sector, biocomputing offers a compelling alternative to traditional, energy-intensive computing systems. As we examine the ethical dimensions of this potential transition, two distinct implications emerge: the environmental responsibility to pursue sustainable computing approaches and the democratizing potential of self-powered computational systems that function without extensive electrical infrastructure.

### Environmental Impact Reduction

Different biocomputing architectures conserve energy through various mechanisms: Benenson et al., eliminated the need for DNA ligase (the enzyme that catalyzes the ligation operation), reducing system complexity by 50% while making the molecular components recyclable^,^ (Shapiro and Benenson, 2006); Nicolau et al.’s biomechanical system used molecular motors (powered by ATP) to convert chemical energy to mechanical energy with near-perfect efficiency, eliminating the need for external power sources (Nicolau et al., 2016) and Tamsir et al.’s bacterial NOR gates showed how cellular networks could perform complex computations using only the energy produced by their normal metabolic functions (Tamsir et al., 2011).

The environmental benefits offered by this energy efficiency remain largely theoretical, stemming from established principles of molecular energetics. For example, Adleman’s experiment showed that DNA computers could approach the theoretical limits of computation imposed by the second law of thermodynamics (Adleman, 1994). Although traditional computing has evolved since 1994, this fundamental advantage of biocomputing persists, suggesting the potential to reduce energy consumption in computational tasks by shifting existing computing infrastructure to energy-efficient biocomputers. The unchecked expansion of the technology sector (driven by the generative AI boom) has increased the global demand for computing power, transformed patterns of electricity use, and accelerated the depletion of natural resources (Commins and Irion, 2025). Technology ethicists are increasingly stressing a moral, societal imperative to reconsider the trajectory of AI (in particular, generative AI) development and its implications for the ecosystem (U. N., 2024)^.^

The environmental implications of increasing computing efficiency become particularly compelling when considering the current trajectory of traditional compute. Today’s data centers consume approximately 1–2% of global electricity (projected to increase to 4% by the end of the decade) and contribute significantly to greenhouse gas emissions (Goldman Sachs, 2024). Theoretical modelling suggests that transitioning complex, operational tasks (e.g., AI search queries) from traditional computing systems to biocomputers that operate near theoretical energy efficiency limits could reduce this environmental impact and reduce the carbon footprint of the technology sector (van Wynsberghe, 2021). However, comprehensive lifecycle assessments of scaled biocomputing systems are a critical gap in current literature. Future research should prioritize quantifying the potential carbon reduction from transitioning appropriate computational tasks to biocomputing architectures. Such assessments would need to account not only for operational energy advantages, but also, for the resource requirements of biological substrate preparation, laboratory conditions, and waste management. Critically, an accurate evaluation of the environmental advantages of biocomputing must integrate not only theoretical efficiencies but also practical lifecycle considerations such as substrate sourcing, biosynthesis of molecular components, and ecological risks associated with biological waste disposal. Addressing these lifecycle impacts requires holistic sustainability assessments, including comparative analyses against traditional silicon-based computing methods. Such comprehensive analyses would ensure more responsible policymaking and identify realistic scenarios where biocomputing delivers genuine environmental benefits.

### Democratization of Computing Resources

Self-powered biocomputing systems like those developed by Nicolau et al., and Tamsir et al., offer considerable promise for democratizing computational access, particularly in resource-limited regions with inadequate electrical infrastructures. However, realizing this potential requires proactive policy measures, including sustained investments in workforce education and local research infrastructure, alongside robust frameworks for equitable technology transfer. Without deliberate intervention, biocomputing risks reinforcing technological divides, with the advanced biological expertise and costly laboratory infrastructure becoming centralized in affluent research environments. Thus, ensuring equitable distribution necessitates targeted capacity-building initiatives and inclusive governance strategies explicitly addressing these socio-economic barriers. Continued research and development in this field point to further improvements in the efficiency of biocomputing systems. Beyond their baseline energy efficiency, biological resource conservation (e.g., by recycling molecular components, per Benenson et al.,) can further buttress the sustainability of these systems. The social implications of environmentally sustainable systems include reduced operational costs (e.g., by minimizing system waste and reducing material requirements) and increased accessibility of computational resources (e.g., by removing the need for expansive electrical infrastructure). Furthermore, the distributed architectures of network-based biocomputing systems (e.g., Tamsir et al.; Didovyk et al.) challenge the prevailing model of centralized computational infrastructure. Unlike traditional data centers that require massive electrical and cooling infrastructure, these bacterial computing networks operate through localized cellular interactions and chemical signalling. This inherent structure suggests the potential for more geographically distributed computational resources, though the social implications of this possibility remain largely unexplored in the literature.

Therefore, biocomputing may theoretically permit greater geographic resource distribution and promote decentralized computing. However, the specialized knowledge base and sophisticated laboratory equipment currently required to operate these systems create barriers to access that could ultimately replicate or exacerbate existing digital divides. Without deliberative policy intervention, the environmental advantages of biocomputing could remain concentrated within elite research institutions and corporations, limiting its democratizing potential. This gap between technical possibility and social reality echoes a distinct challenge observed across biotechnology domains: specialized expertise often leads to centralization despite technical possibilities for decentralized implementation (Keulartz & van den Belt, 2016). Future governance frameworks should explicitly address questions of knowledge transfer, capacity building, and ownership models to ensure that the unique benefits introduced by biocomputing translate to broader *social* benefits, rather than reinforcing existing patterns of technological inequality. However, biosafety risks become increasingly salient if access to computational bacterial populations is broadened; as such, any policy intervention must explicitly address gaps in biosecurity knowledge and regulatory transfer for decentralized systems.

## Error Rate in Biocomputing: Accuracy Metrics and Clinical Implications

Adleman’s experiment revealed unique challenges in the computational accuracy of molecular computing systems: governance frameworks built for traditional electronic computing systems are insufficient in addressing the inherently probabilistic nature of molecular computation, as well as the natural error introduced through physical factors (e.g., humidity, contamination, degradation, and mechanical vibration). While traditional computing relies on deterministic error-checking and verification, biocomputing operates within a fundamentally different paradigm in which certainty must give way to statistical confidence. This paradigm shift requires reimagining common conceptions of computational accuracy, not merely adapting existing regulations, particularly when biocomputing systems directly impact patient care through diagnostic and therapeutic applications. Given the probabilistic nature and reactional uncertainty in biocomputing diagnostics for example, addressing potential biases and inaccuracies will be crucial, as emphasized by similar issues in traditional AI-based medical systems (Challen et al., 2019)^.^

Emerging biocomputing technologies in clinical contexts will also necessitate robust regulatory oversight, including post-market surveillance mechanisms, clear reporting requirements for computational inaccuracies, and transparent disclosure to patients regarding the probabilistic nature of results. Ethical guidelines tailored explicitly to biocomputational diagnostic and therapeutic interventions will strengthen patient safety and enhance public trust.

### Regulatory Frameworks for Accuracy

Several strategies have been proposed to address biocomputing error rates. For example, Benenson et al., address concerns regarding the error rate of scaled-up computation by circumventing the DNA ligation step altogether, thereby reducing whole-system complexity and removing one source of error (Benenson et al., 2004). Other potential error correction strategies emerge in the literature. Two such strategies are: (1) incorporating error correction mechanisms into algorithmic development upstream, and (2) building error-resistant (rather than self-correcting) systems. An example of the former is the work of Winfree and Bekbolatov (2004), who promote incorporating “proofreading tile sets” into algorithmic construction, such that the system itself is built with baseline error correction mechanisms in place (Winfree & Bekbolatov, 2004). An example of the latter is the Liverpool-Warwick molecular computing group (part of the European Molecular Computing Consortium), whose work in implementing DNA computing models requires translating the molecular operations of these models into Boolean circuits through error-resistant strand removal (EMCC, n.d.).

Despite these efforts in proactive error mitigation, significant regulatory gaps regarding *post hoc* protections remain. New governance frameworks must establish acceptable error thresholds for different applications, with stricter standards for critical use cases (e.g., life-sustaining or life-saving interventions). One way to address these gaps could be through graduated risk classification systems for biocomputing applications based on the severity of potential harms arising from computational errors. Given the hybrid nature of biocomputing systems, which combine biological substrates with computational tasks, existing regulations from both biotechnology and computational domains may require integration and harmonization. This interdisciplinary regulatory integration is necessary to avoid fragmentary oversight and to establish cohesive standards for system accuracy, reliability, and accountability. Regulatory bodies might consider developing hybrid standards tailored to biocomputational technologies, and should: clearly define acceptable risk levels; specify validation protocols for different biological substrates; outline minimally acceptable conditions for the storage and maintenance of these substrates; and ultimately, standardize methodologies for error correction and validation.

### Clinical Implications

Clinical implications of biocomputing error rates extend beyond theoretical concerns, directly impacting patients and, thus, demanding careful ethical consideration. In Benenson et al.,’s diagnostic application, a false positive error (where a product forms but encodes an invalid solution) would result in the release of an ssDNA molecule capable of altering the gene expression of a healthy individual; a false negative error (where no product forms despite a valid path existing in theory) would prevent the release of a therapeutic molecule in an individual with the disease of interest (Benenson et al., 2004). Thus, in such cases, error rates must be carefully managed to promote patient safety.

Among other architectures, like Didovyk et al.,’s network-based distributed classifier system, individual cellular errors are leveraged as part of the system’s design (Didovyk et al., 2015). While individual bacterial cells act as “weak classifiers” and are, by definition, error-prone, the collective population response provides robust classification through consensus (Didovyk et al., 2015). This approach mirrors natural biological error response, leveraging innate checks and balances. For example, the immune system uses a similar “network-based” error response, whereby cellular population-level accuracy produces an immune response despite individual cell fallibility. Further research could explore networking biochemical, biomechanical, and bioelectric computing architectures to provide a systemic “fail-safe” in cases of large-scale computation. This approach requires embracing statistical confidence rather than binary certainty—a fundamental paradigm shift from deterministic models. With robust statistical frameworks, however, a probabilistic approach can achieve reliability levels appropriate for many applications.

In contrast to biocomputing errors, traditional computing errors rarely have immediate physiological consequences. Thus, emerging biocomputing governance frameworks must establish acceptable error thresholds and comprehensive risk stratification protocols considering disease severity, treatment urgency, intervention reversibility, and alternative diagnostic options. Furthermore, unlike traditional medical devices with well-established safety classifications, molecular diagnostic devices are hybrid systems that combine computational and biological risks, necessitating new regulatory approaches that address this convergence as an independent feature of these systems rather than attempting to create a regulatory patchwork between existing analogues.

## Information Storage in Biocomputing: Patentability, Data Ownership, and Regulatory Oversight

Legal frameworks designed for static, non-living data storage technologies collapse when confronted with biological information systems that can replicate, evolve, and interact with other living systems. At this new intersection of information technology and biology, we identify three critical legal gaps: determining the boundaries of patentability in semi-natural molecular storage systems, establishing ownership rights when stored information reproduces autonomously through cell division, and developing comprehensive regulatory approaches for biological data repositories.

### Patentability Challenges

The patentability of biological storage systems confronts existing legal frameworks due to their reliance on naturally occurring molecules and processes. Patenting engineered biological arrangements demands clear delineation between naturally occurring biological sequences and patentable modifications, a differentiation fraught with potential ambiguities and legal disputes, as exemplified by CRISPR technologies. For example, Tamsir et al.,’s bacterial NOR gates use engineered schemas of naturally functioning cellular mechanisms (Tamsir et al., 2011), raising important questions about what aspects of biological storage systems can reasonably be protected under intellectual property law. Innate molecular sequences and biological processes underlying engineered arrangements are common to living organisms and thus cannot be patented. However, their engineered arrangements, modifications (e.g., manipulated sequences), and unique data storage or encoding methods *are* patentable. Therefore, regulatory bodies may need to establish robust, transparent criteria that precisely define patentable biological innovations, distinguishing engineered manipulations from naturally occurring biological processes. This approach will not only reduce litigation risks but also clarify intellectual property rights, facilitating responsible innovation and equitable commercial development in biocomputing technologies.

Established biotechnology patents, like CRISPR-Cas9, have faced similar criticism, particularly regarding ethical and governance concerns about genetic manipulation and the limits of human control over biological evolution (Doudna & Sternberg, 2017; CRISPR, 2023). The CRISPR patent disputes between the Broad Institute and the University of California exemplify these challenges, with litigation centring on whether applications of CRISPR-Cas9 in eukaryotic cells represented a non-obvious extension of its naturally occurring bacterial function (Egelie et al., 2016). Much like CRISPR, distinctions may *technically* be drawn between naturally occurring processes and patentable innovations in biocomputing; however, these distinctions may appear arbitrary in the face of existing regulation. While it is reasonable to expect international regulatory bodies to differ in patent oversight frameworks, regulatory inconsistencies create significant challenges for commercial development.

### Data Ownership in Self-Replicating Systems

Data ownership also becomes particularly complex when dealing with self-replicating storage systems. If one begins storing data in biological substrates, some biocomputing approaches, like Didovyk et al.’s bacterial classifier system, raise new questions regarding the implications of cellular division. The nucleoid of prokaryotic bacterial cells contains DNA, which, as described in §6.1, can be used to store information. As bacterial populations storing computational data replicate, questions may arise regarding who rightfully owns the information in the daughter cells, as well as some further normative questions. For example, it is unclear whether data replication during cell division should be treated as data replication (and, thus, whether HIPAA protections are violated if these data are used in medical contexts). It is also unclear how genetic mutations (which occur probabilistically and cannot be fully controlled) would alter stored data, if at all, nor how to treat these data modifications if they do occur. These unique characteristics of self-replicating biological storage systems may also raise public concerns similar to those encountered in synthetic biology, where this perceived “unnaturalness” of engineered organisms has generated societal resistance (Dragojlovic & Einsiedel, 2013)^.^

Thus, traditional legal protections for digital data ownership are insufficient for addressing the characteristics of biological storage systems capable of autonomous replication and evolution. Establishing transparent data protection frameworks that clearly communicate ownership rights and biological safeguards could help address societal concerns while fostering public trust in biocomputing applications. Future regulatory frameworks must proactively anticipate scenarios of genetic drift, data mutation, and unauthorized biological reproduction. To manage these challenges effectively, it may become necessary to implement continuous ownership tracking through genetic watermarking or digital signatures embedded at the molecular level, ensuring legal clarity and enforceability even amidst biological complexity.

### Regulatory Frameworks for Biological Data Storage

Due to these considerations, the regulatory landscape for biological data storage remains largely undefined. Some existing frameworks for genetic engineering and biosafety protocols may apply. However, specific regulations for biological data storage systems have not yet been established. Storing information on DNA remains mainly theoretical, with recent developments (e.g., Zhang et al.’s “epigenetic bits framework”) aiming to reduce existing resource constraints and improve the practical applicability of these storage systems (Zhang et al., 2024). Existing regulatory gaps may include biosafety regulations (e.g., for handling sensitive biological data), data security regulations (e.g., for handling data storage and ownership in self-replicating systems), and medical device regulations (e.g., for handling the appropriate application of these systems and promoting patient safety). These regulatory gaps are particularly evident in biochemical storage systems like Benenson et al.,’s autonomous molecular computer, which crosses each of these regulatory boundaries. Benenson et al.’s diagnostic system (a) stores medical data (e.g., diagnosis) in biological form; (b) processes sensitive health information (e.g., uses diagnostic data to action a therapy or placebo); and (c) generally operates as a diagnostic tool (for which medical device regulation must apply) (Benenson et al., 2004). Comprehensive legal frameworks for biocomputing must address the unique challenges introduced by these systems while weighing stakeholder interests.

Policy efforts should prioritize the development of specialized regulatory agencies or oversight bodies dedicated to biological data storage and biocomputing. These agencies could coordinate cross-sectoral standards for biosafety, data security, and intellectual property, ensuring comprehensive and unified governance. International harmonization of these regulatory approaches is also crucial, as biocomputational technologies rapidly transcend national boundaries, necessitating collaborative international policy frameworks.

## Conclusion and Future Considerations

In this article, we have identified several new GELSI of biocomputing that extend beyond those identified in and addressed by existing regulatory frameworks for traditional computing methods. While the GELSI analysis in this article discusses many considerations not exclusive to biocomputing (e.g., patient safety and intellectual property, for instance), the field presents its distinct challenges. By examining four key characteristics—scalability, energy efficiency, error rate, and information storage potential—we identified several salient implications that may arise, meriting greater attention as the field advances. As described, our GELSI analysis is non-exhaustive since we aimed to identify areas with sufficient evidence to warrant regulatory action, particularly given the innovative potential and rapid expansion of this field.

Existing legislative frameworks lack the comprehensiveness required for the ethical adoption of biocomputing technologies. For example, contemporary regulatory structures governing biotechnology, data security, and medical device implementation are insufficient in addressing questions of safe scaling, informational storage on molecular substrates, and biocomputationally-mediated therapies. This regulatory gap must be addressed to safely deploy biocomputing technologies across sectors, particularly for personalized medicine.

Future work may explore developing targeted regulatory frameworks that specifically address the GELSI arising from the four unique characteristics of biocomputing we identify, thereby balancing innovation with appropriate safeguards. These frameworks may include: (1) specialized verification protocols that accommodate probabilistic computation, (2) biosafety standards for scaled biological systems, (3) clear intellectual property guidelines for biological computing components, and (4) risk stratification approaches for clinical applications. Furthermore, while our analysis has considered how these GELSI arise across four popular biocomputing architectures (e.g., biochemical, biomechanical, bioelectrical, and network-based), it is worth acknowledging the emerging field of organoid intelligence (OI) as a related, yet distinct, domain with its own set of implications. The use of neural organoids in OI introduces additional complexities related to consciousness, sentience, and neural development that extend beyond the scope of this paper. Early work in this area has been explored, but additional regulatory safeguards will be necessary as OI biocomputing is better understood.

Our aim in this article was to provide a sufficiently comprehensive analysis of the GELSI implications of biocomputing while identifying specific conceptual and regulatory blind spots. Moving forward, a principled approach to governance will require not only reactive oversight but also anticipatory ethical design. Policymakers must recognise that biocomputing blurs traditional distinctions between hardware and wetware, between code and cell. This collapse of categories demands that ethical and legal frameworks evolve at pace—reflexive, interdisciplinary, and globally harmonised. The responsible integration of biocomputing will depend not on retrospective regulation alone but on our capacity to embed foresight and fairness into the very architectures of living computation.

## Data Availability

Not applicable.
